# Effects of axial malrotation on posterior tibial slope measurement: a digitally reconstructed radiograph study enabling automated quality assessment

**DOI:** 10.1186/s43019-026-00303-x

**Published:** 2026-01-27

**Authors:** Jaeseok Park, Andreas Persson, R. Kyle Martin, Eivind Inderhaug, Sung Eun Kim, Sangyoon Kim, Donghyuk Kwak, Du hyun Ro

**Affiliations:** 1 Seoul National University College of Medicine, Seoul, Republic of Korea; 2 Oslo Sports Trauma Research Center, Norwegian School of Sports Science, Oslo, Norway; 3 Department of Arthroscopy and Sports Medicine, Oslo University Hospital Aker, Oslo, Norway; 4 Department of Orthopedic Surgery, University of Minnesota, Minneapolis, MN USA; 5 Department of Orthopedic Surgery, CentraCare, Saint Cloud, MN USA; 6 Department of Clinical Medicine, University of Bergen, Bergen, Norway; 7 Haukeland University Hospital, Bergen, Norway; 8 Department of Orthopedic Surgery, Seoul National University College of Medicine, Seoul National University Hospital, 101 Daehakro, Jongno-Gu, Seoul, 110-744 Republic of Korea; 9 National Strategic Technology Research Institute, Biomedical Research Institute, Seoul National University Hospital, Seoul, Republic of Korea; 10 Department of Biomedical Informatics, Harvard Medical School, Boston, MA USA; 11 CONNECTEVE Co., Ltd, Seoul, Republic of Korea

**Keywords:** Posterior tibial slope, Digitally reconstructed radiograph, Axial malrotation, Lateral knee radiograph, Artificial intelligence

## Abstract

**Background:**

Accurate measurement of posterior tibial slope (PTS) is highly sensitive to axial malrotation of the knee during acquisition, but its impact has not been systematically quantified across different anatomical variations. This simulation study aimed to quantify the effect of axial malrotation on PTS using digitally reconstructed radiographs (DRRs) and suggest a practical marker for filtering out low-quality images with excessive malrotation.

**Materials and methods:**

A total of 55 preoperative computed tomography (CT) scans from January 2021 to December 2024 in a single, tertiary hospital were retrospectively reviewed. DRRs were generated from those scans to simulate lateral knee radiographs with malrotation ranging from −12° to +12° relative to an anatomically aligned baseline. An artificial-intelligence (AI)-based tool automatically measured PTS on each DRR, with agreement evaluated using intraclass correlation coefficient (ICC). PCDR was calculated from femoral contours and analyzed for correlation with malrotation angles and resulting PTS measurement error.

**Results:**

AI-based PTS measurements on DRRs showed good agreement with expert annotations (ICC = 0.78, 95% CI 0.73–0.82). PTS increased linearly with internal rotation, with each 1° of rotation resulting in approximately 0.2° change in PTS (*R*^2^ = 0.43, *p* < 0.01). Errors exceeded 1° when malrotation surpassed ±6°. PCDR was strongly correlated with malrotation angle (*R*^2^ > 0.98, *p* < 0.001) and achieved fair discriminative performance as a binary classifier for > 1° PTS error [area under the receiver operating curve (AUROC) = 0.77].

**Conclusions:**

CT-derived DRRs combined with AI analysis showed that PTS measurement error proportionately increased with axial malrotation. Identifying and excluding radiographs with excessive rotation improves the reliability of slope-based assessments and supports more accurate surgical planning.

**Level of evidence:**

III, retrospective cohort study.

**Supplementary Information:**

The online version contains supplementary material available at 10.1186/s43019-026-00303-x.

## Background

The posterior tibial slope (PTS), defined as the inclination angle of the tibial plateau relative to the long axis of the tibia, is clinically important owing to its significant influence on knee biomechanics and stability. Variations in PTS affect the anterior–posterior motion of the knee, placing increased strain on ligaments such as the anterior cruciate ligament (ACL) and posterior cruciate ligament (PCL) [1, 2]. An increased PTS is strongly associated with a higher risk of ACL injury and graft failure (after ACL reconstruction), with PTS of ≥ 12° associated with increased risk of subsequent ligamentous injuries or recurrent instability [3–7]. Furthermore, PTS plays a critical role in surgical planning for knee procedures, including high tibial osteotomy [8] and total knee arthroplasty [2, 9]. Even minor unintended alterations in PTS during these procedures can lead to changes in ligament tension, compromised joint stability, and suboptimal postoperative biomechanics.

Given the important role of PTS in the surgical decision-making process, accurate measurement of PTS is essential. Lateral knee radiographs are most commonly used in clinical practice, employing methods that measure the angle between the tibial plateau and the tibial shaft [10]. Despite their simplicity, PTS measurements require precise radiographic techniques, and slight variations in positioning or method can significantly alter results [11–13]. To date, no study has systematically evaluated how PTS measurements vary across a continuous range of axial malrotation using real patient anatomy. Owing to ethical and practical difficulties in acquiring multiple lateral knee radiographs from the same patient at incrementally different rotational positions, recent studies have utilized sawbone models to obtain measurements at discrete positions [12, 14] or one rotated radiograph from each patient with an unclear amount of malrotation [13].

Digitally reconstructed radiographs (DRRs), generated from computed tomography (CT) images [15, 16], offer a practical solution by enabling precise simulation of radiographs under systematically varied axial rotation. Unlike physical models or opportunistic clinical images, DRRs allow controlled manipulation of knee positioning using real anatomical data without additional radiation exposure. These simulated radiographs can be directly analyzed by a previously developed and externally validated AI tool for automated PTS measurement [17, 18], enabling large-scale, standardized evaluation of measurement variability. Inversely, the amount of malrotation present in a radiograph can be assessed from overlap of the medial and lateral condyles of femur.

This study aimed to (1) determine how axial malrotation in lateral knee radiographs affects measurement error of PTS using a standardized simulation framework, and (2) develop and validate surrogate image marker for evaluating image quality and potential PTS measurement error. We hypothesized that PTS measurements would change in a predictable linear trend with increasing internal or external axial malrotation, and a simple radiographic marker could serve as a threshold-based image quality metric for filtering radiographs with potential PTS error exceeding 1°.

## Methods

### Study design

This was a diagnostic simulation study (level of evidence III) using digitally reconstructed radiographs generated from CT scans to systematically evaluate the effects of controlled axial malrotation on PTS measurement error. This study was approved by the Institutional Review Board (IRB, no. 2503–163-1625) of the authors’ institute. Informed consent was waived owing to the retrospective nature of the study.

**Fig. 1:**
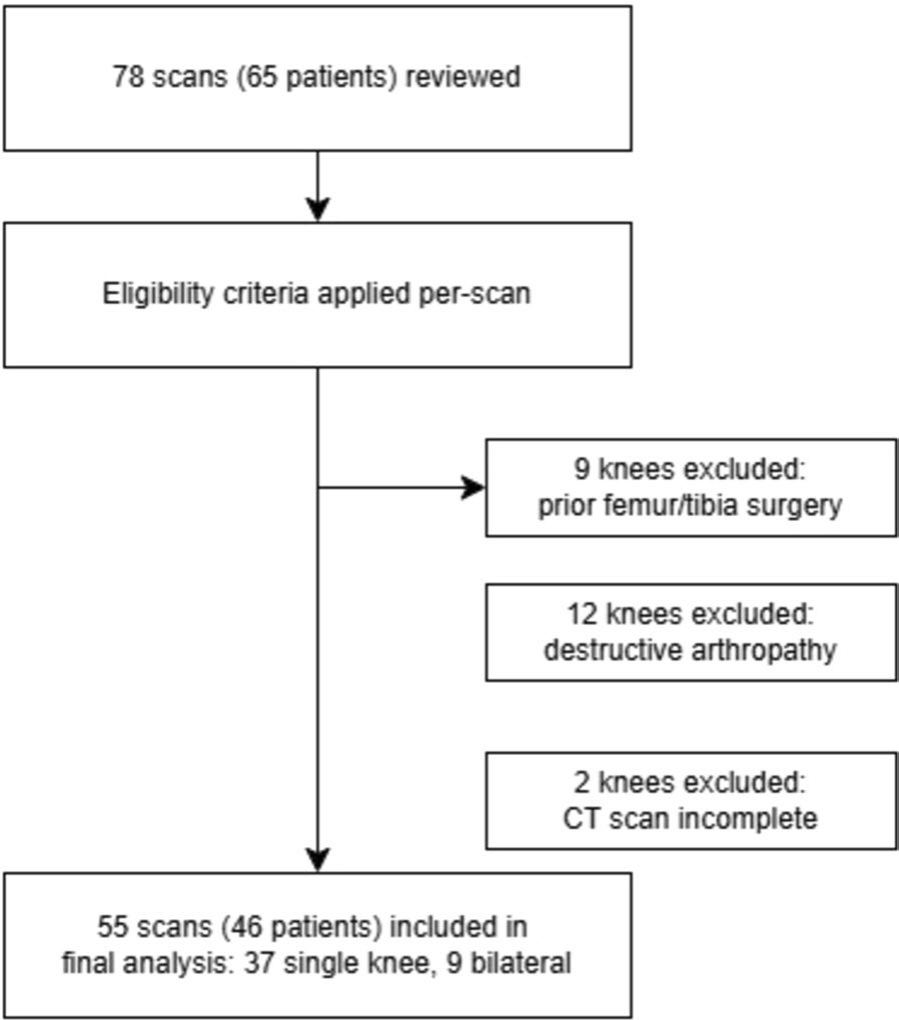
Flowchart of data inclusion and exclusion

We retrospectively reviewed all 65 patients who underwent lower limb computed tomography (CT) scans for patient-specific instrumentation (PSI) total knee arthroplasty (TKA) at our institution between January 2021 and December 2024. All CT scans in our study were acquired using a uniform protocol, with pixel spacing, slice thickness, and spacing between slices of 0.4883 mm, 1.0 mm, and 0.5 mm, respectively. This CT protocol was selected for its high slice resolution, which is essential for generating high-quality DRRs [19]. We excluded knees with evidence of (1) prior surgery involving the femur or tibia, (2) destructive arthropathy or large osteophytes obscuring the tibial plateau contour, (3) incomplete images. A total of 55 knees from 46 patients met all criteria and were included in the final analysis (Fig. 1). Bone segmentation was performed manually on femur and tibia from each CT scan by an experienced knee surgeon.

### CT volume rotation and generation of digitally reconstructed radiographs

For accurate diagnosis of knee joint pathologies, lateral knee radiographs must show sufficient overlap between the medial and lateral femoral condyles [12, 20]. Prior to simulating malrotation, the original CT volume was rotated to establish an anatomically ideal alignment, defined by complete overlap of the femoral condyle contours on lateral projection and zero inclination of the tibial plateau in the coronal plane. This configuration was designated as the “aligned volume” and served as the baseline for all subsequent analyses. We observed that standardizing these conditions ensured measurement consistency and minimized potential errors in automatic PTS measurement by a pretrained model (see “Automatic posterior tibial slope measurement” section).

The two- step process (coronal rotation and axial rotation) of generating the aligned volume is summarized in the Supplementary Information. First, the tibia was rotated in the coronal plane to eliminate mediolateral tilt. This step ensured that the medial and lateral tibial plateaus were aligned in the sagittal projection. Although coronal tilt from mechanical alignment or patient positioning can also affect PTS measurement, we intentionally removed this variability to isolate the effects of axial malrotation under standardized slope conditions.

**Fig. 2:**
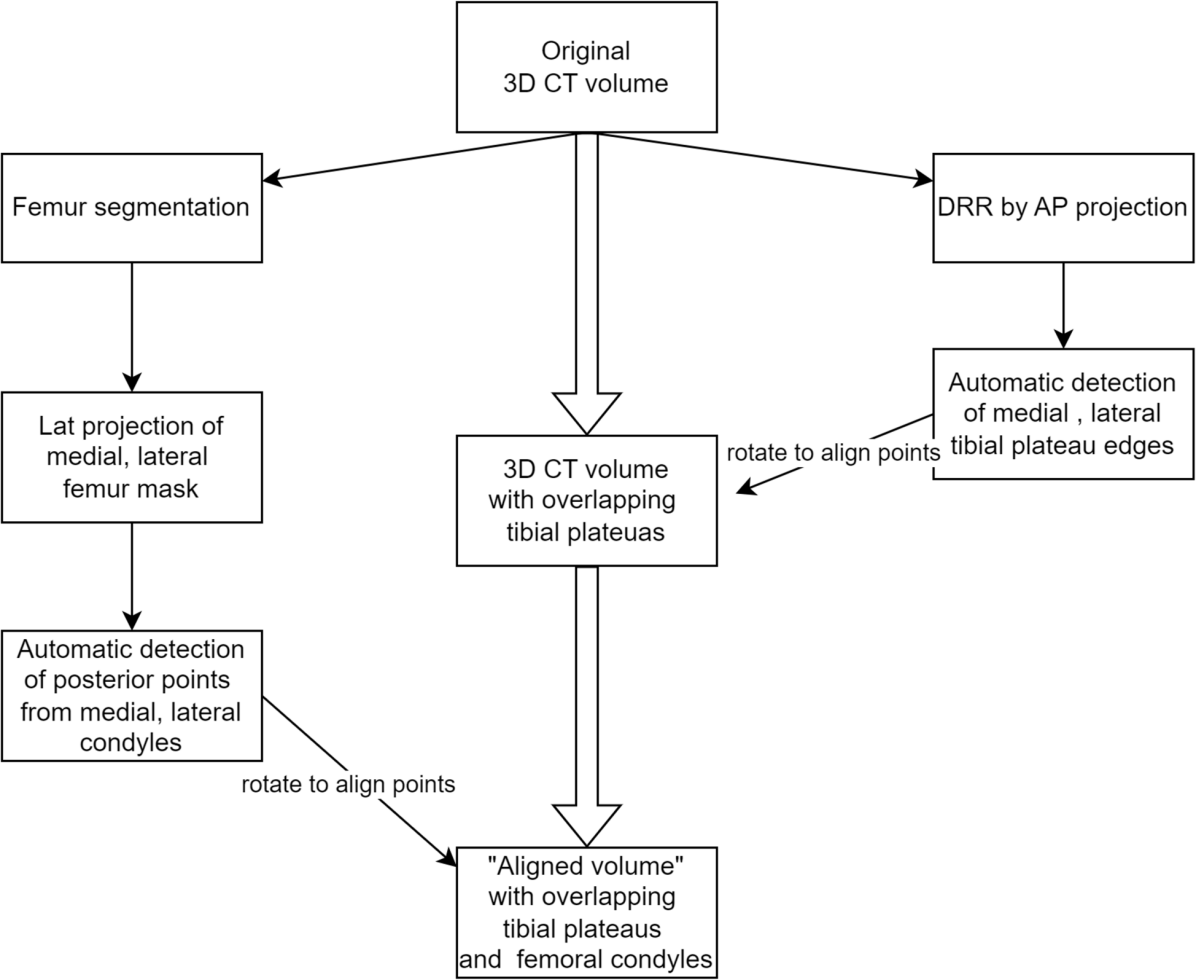
Flowchart of processing original computed tomography (CT) image into aligned volume

Second, axial rotation was applied to superimpose the medial and lateral femoral condyles on lateral projection, using posterior condylar “posterior points” derived from condyle-specific two-dimensional (2D) masks referenced to a common anterior cortical line. Although the posterior condylar axes of the femur and tibia may not be perfectly aligned, we selected femoral condyle overlap as the reference for “true lateral” projection on the basis of its clinical standardization and reproducibility. This reference served as a fixed baseline for quantifying relative changes in PTS under controlled axial malrotation. The overall process of automated CT volume alignment is summarized as a flowchart in Fig. 2, with additional visualizations of specific steps in Supplementary Fig. 2.

With the aligned volume as baseline, we performed rigid axial rotations about the CT scans’ longitudinal axis, (positive = internal rotation, negative = external rotation in 2° increments across −12°, −10°, …, +10°, +12°). Thus, each knee yielded 13 standardized positions. Sign conventions and axes remained consistent across all cases.

For each rotated volume, digitally reconstructed radiographs (DRRs) were generated using the All Scale Tomographic Reconstruction Antwerp (ASTRA) Toolbox [21] with cone-beam geometry (source–detector distance: 1200 mm; source–patient distance: 1120 mm). A 240 × 240 × 400 mm^3^ volume centered on the knee joint was extracted and resampled to 0.5 mm isotropic spacing. CT data were preprocessed using a bone window (center: 400 HU; width: 1500 HU) and converted to attenuation coefficients using a nonlinear mapping optimized for bone contrast enhancement (*μ*_water_ = 0.02 mm^−1^ at 66 kVp). The forward projection produced 2700 × 1800 pixel lateral radiographs (detector pixel size 0.148 mm). To simulate realistic X-ray appearance, images underwent gamma correction (*γ* = 0.7), Poisson noise addition (10,000 photon scale), Gaussian noise (*σ* = 0.002), and Gaussian blur (*σ* = 0.5 pixels). Final images were saved as 16-bit grayscale with values in the range of 0–4095. The DRR reconstructed from the aligned volume was defined as the “true lateral.”

### Automatic posterior tibial slope measurement

**Fig. 3:**
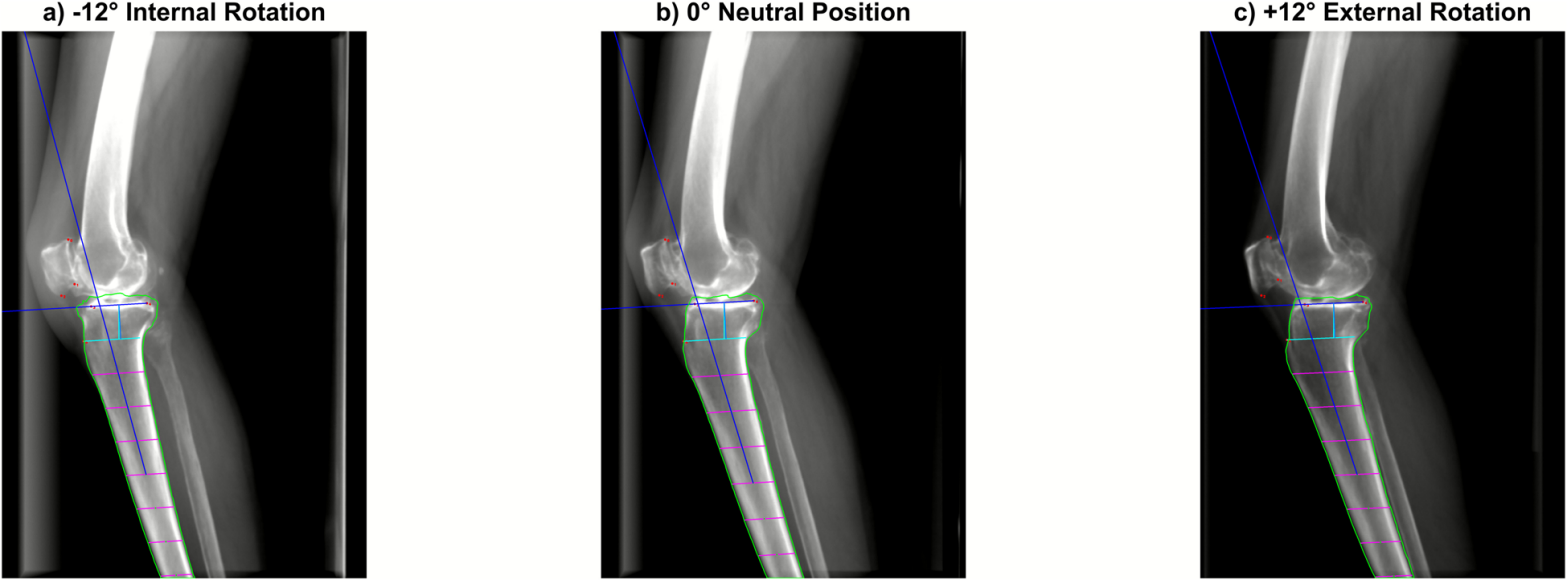
Example of DRR generation and PTS measurement with varying rotation

PTS was automatically measured on each DRR using a previously validated deep learning model trained on 9277 lateral knee radiographs from a single tertiary hospital between 2009 and 2019 [17, 18]. Briefly, a DEKR-based landmark detector localized six anatomical landmarks, and a segmentation-based algorithm estimated the tibial shaft axis; PTS was then defined as the angle between the shaft axis and the medial plateau tangent as per our prior work. The model was trained on 9277 radiographs, validated on 500, and tested on 230 independent images. In our previous studies, the model demonstrated excellent agreement with manual measurements using the landmark-based method (ICC = 0.91–0.92 overall), and perfect intra-observer reliability (ICC = 1.00). It also showed good external validity when applied to an independent dataset of 289 lateral knee radiographs from ACL-injured patients, achieving a good inter-rater ICC of 0.73–0.80 compared with manual measurements and maintaining perfect intra-rater reliability (ICC = 1.00). ICC values were interpreted as: < 0.50 = poor, 0.50–0.75 = moderate, 0.75–0.90 = good, and > 0.90 [22]. Example generated DRRs and overlaid visualizations of automatic PTS measurement are shown in Fig. 3.

### Manual posterior tibial slope measurement

To validate the reliability of automatic measurements in DRRs, PTS was manually measured for DRRs with selected rotation angles of −8°, −4°, 0°, 4°, 8°, resulting in 275 human measurements (5 images each for 55 patients). It must be noted that, while automatic measurements were done in 13 images per each patient (−12°, −10°… 10°, 12°) to carefully analyze the effect of even small angle changes in PTS measurements, manual measurements and interobserver reliability analyses were only done in a subset of these angles per image as this was sufficient to assess reliability of AI measurements. Manual PTS was measured as the angle between the medial tibia plateau tangent and tibial shaft axis. The tibial shaft axis was calculated by connecting midpoints of tibia shaft located at two levels below the joint line, where each level was defined by 2 and 4 multiples of the perpendicular distance from the tibial joint line to the proximal tibial tuberosity. This definition of tibial shaft axis corresponds to that used in the automatic model, and is identical to the one in our previous studies [17]. The measurements were performed by an orthopedic knee surgeon who recently completed residency and knee fellowship training. The intraclass correlation coefficient (two-way mixed effects, absolute agreement, single measures) was calculated between automatic and manual measurements.

### Calculation of posterior condyle distance ratio (PCDR)

**Fig. 4:**
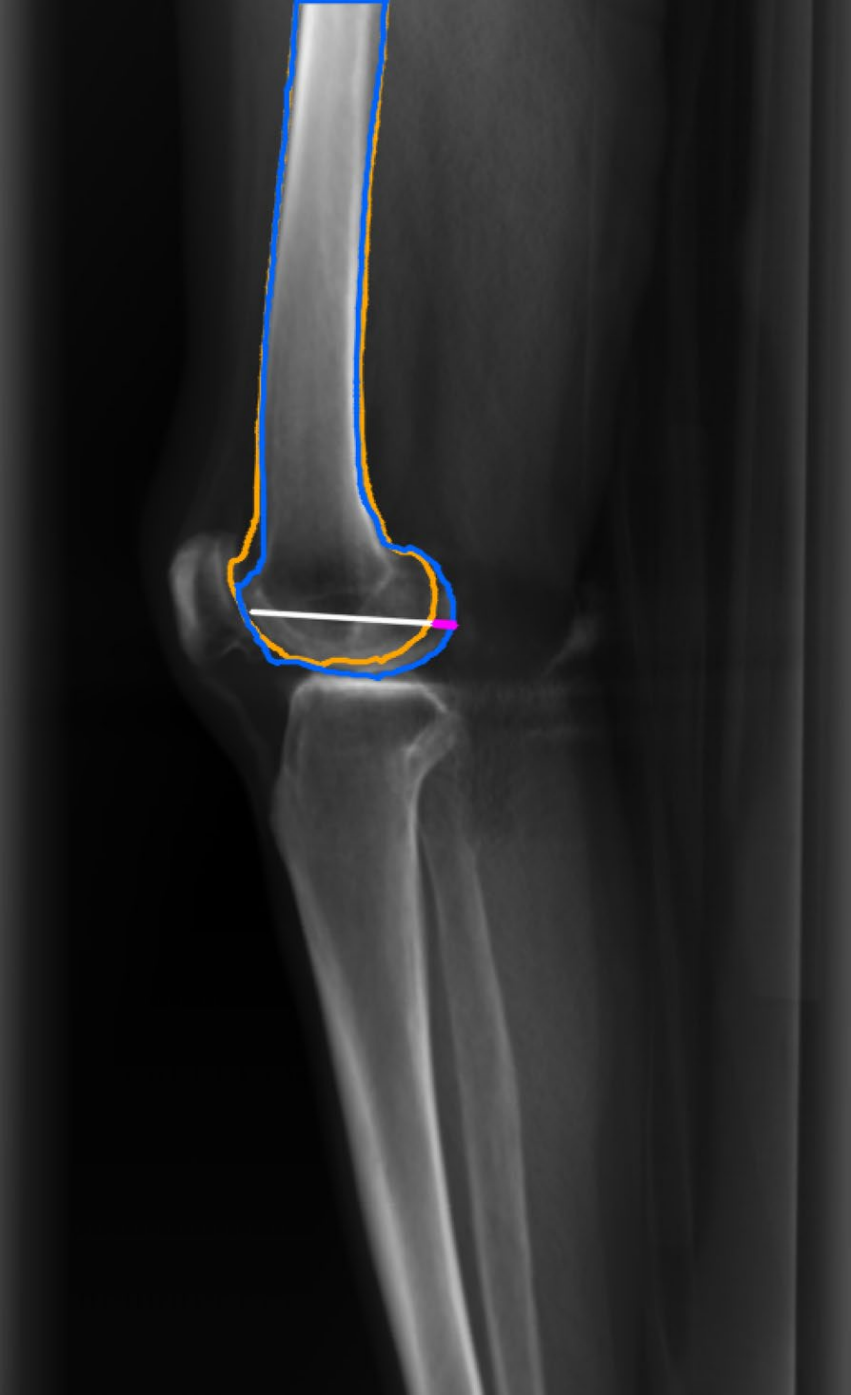
Example of PCDR measurement from DRRs

Tje posterior condyle distance ratio (PCDR) quantifies axial malrotation on lateral views as the normalized posterior femoral condyle displacement. Posterior points of the medial and lateral condyles were located with respect to a common anterior cortical line derived from the full femur mask. The posterior condyle distance was the absolute anterior–posterior offset between the two posterior points; this distance was normalized by the total anterior–posterior femoral joint width (distance from the anterior cortical line to the posterior point on the full mask), yielding a unitless, absolute ratio (Fig. 4).

$$\mathrm{PCDR}= \left|\Delta \text{posterior points of medial and lateral condyle}\right|/(\text{AP width of distal femur})$$

In the aligned volume used to generate the true lateral projection, medial and lateral femoral condyles were fully superimposed, yielding a baseline PCDR of zero. Thus, PCDR was used as a surrogate marker for the degree of axial malrotation in subsequent rotated images. While prior studies have analyzed posterior femoral condyle displacement as a measure of malrotation, this study introduces normalization by femoral condyle width to account for interpatient variation. The process of automatic posterior condyle distance ratio calculation and a semi-automated tool to work on clinical radiographs is further explained in Supplementary Information.

PTS is measured on multiple DRRs with varying amounts of rotation. PTS is defined as the angle between tibia shaft axis and medial plateau tangent (both represented as blue lines), calculated by keypoint detection and segmentation.

PCDR can be calculated by the length of pink segment divided by the sum of lengths of pink and white segments. The white line segment represents AP width of smaller condyle, and the pink segment represents the distance between posterior points of medial and lateral condyles. Blue and orange contours each represent the contours of medial and lateral femur.

### Statistical analysis

We used Student’s *t*-test to determine whether statistically significant differences existed in PTS measurements from true laterals and rotated images. We performed separate paired *t*-tests comparing PTS measurements from each malrotation angle (−12°, −10°… 10°, 12°) with baseline (0° or aligned). Because this involved multiple comparisons (13 total tests), we applied a Bonferroni correction. The nominal alpha level was set at 0.05, and thus the corrected significance threshold became *α*_corrected_ = 0.05/13 ≈ 0.0038. All *p*-values below this threshold were considered statistically significant.

A linear mixed-effects model was employed, with rotation angle as a fixed effect and interpatient variation as a random effect, to evaluate the linear relationship between rotation angle and measurement error from true laterals across different rotation angles and patients. Marginal *R*^2^ was calculated according to the method suggested by Nakagawa and Schielzeth [23] to quantify the proportion of variance explained solely by the fixed effect.

To analyze the relationship between malrotation angle and the posterior femoral condyle distance ratio, linear regression constrained through the origin was performed, based on the assumption that the condylar distance should be zero under perfectly aligned conditions. Data were stratified by direction of rotation into internal (positive) and external (negative) groups. Receiver operating characteristic (ROC) analysis was performed to evaluate the clinical utility of PCDR as a binary classifier for identifying radiographs with clinically significant PTS error. While there is no universally accepted margin of error for PTS measurement, a 1° threshold was chosen to define clinically significant PTS error on the basis of clinical studies showing that even small deviations in PTS can meaningfully affect graft tension, joint stability, and postoperative outcomes, particularly in ACL reconstruction and high tibial osteotomy procedures [3, 4]

## Results

A total of 55 knees from 46 patients were included in the study. Mean age of the cohort was 73 years, and the mean body mass index (BMI) was 27 ± 2.7 kg/m^2^. Detailed patient characteristics are summarized in Table 1.

**Table 1:** Patient characteristics

Category	Count/average
Age [years]	72.7 (4.9)
Sex, male/female	5/41
Height [m]	1.52 (0.06)
BMI [kg/m^2^]	26.6 (2.7)
Ethnicity	
Korean	46 (100%)
Knee side	
Both knees	11
Only right knee	19
Only left knee	14

**Fig. 5:**
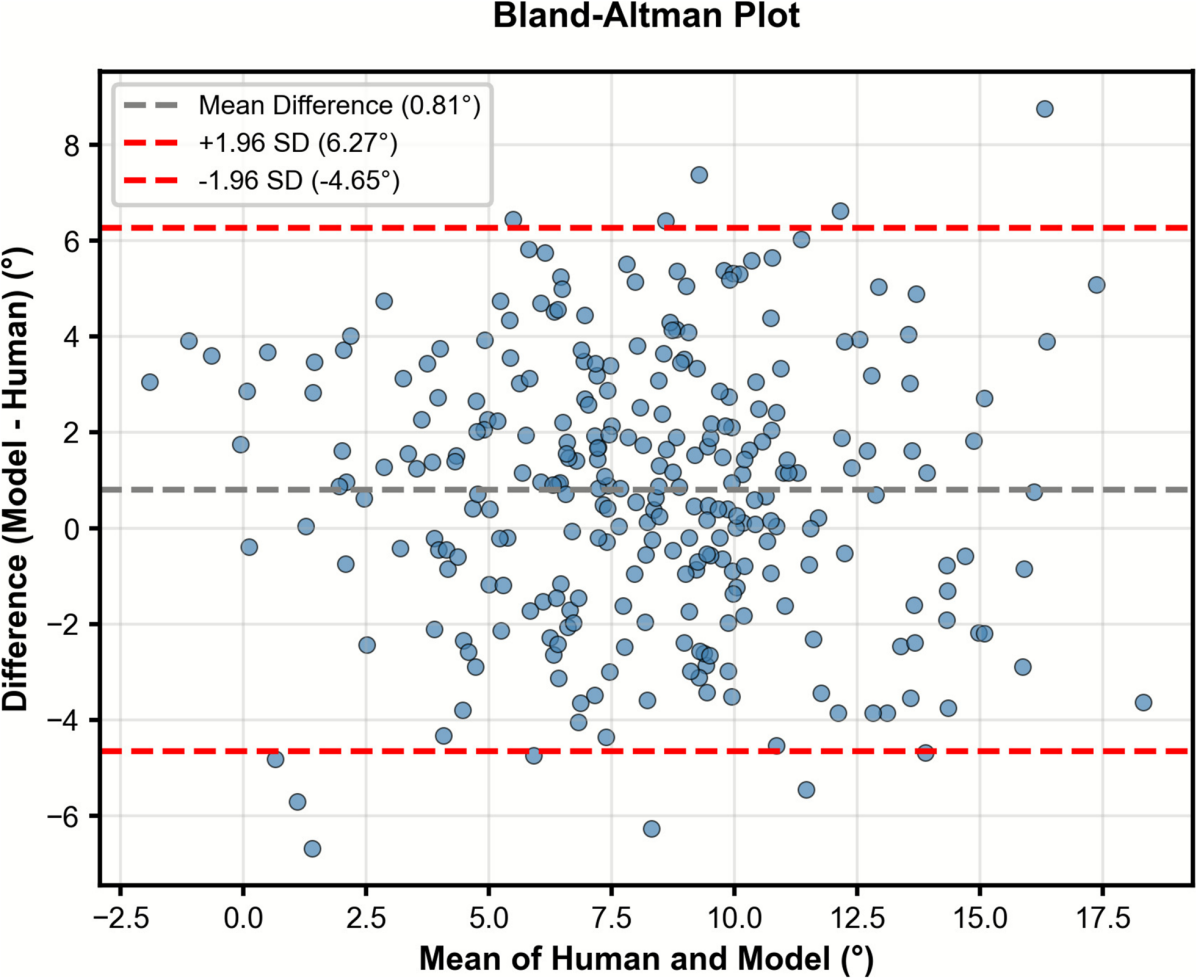
Bland–Altman analysis of PTS measurements on DRRs by AI model and orthopedic specialist

Automatic PTS measurements on DRRs showed good agreement with expert annotations, with an ICC of 0.78 (95% confidence interval 0.73–0.82). Bland–Altman analysis demonstrated a mean difference of 0.81°, with 95% limits of agreement ranging from −4.65° to 6.27°, indicating minimal bias and acceptable agreement across the range of measurements. (Fig. 5).

**Fig. 6:**
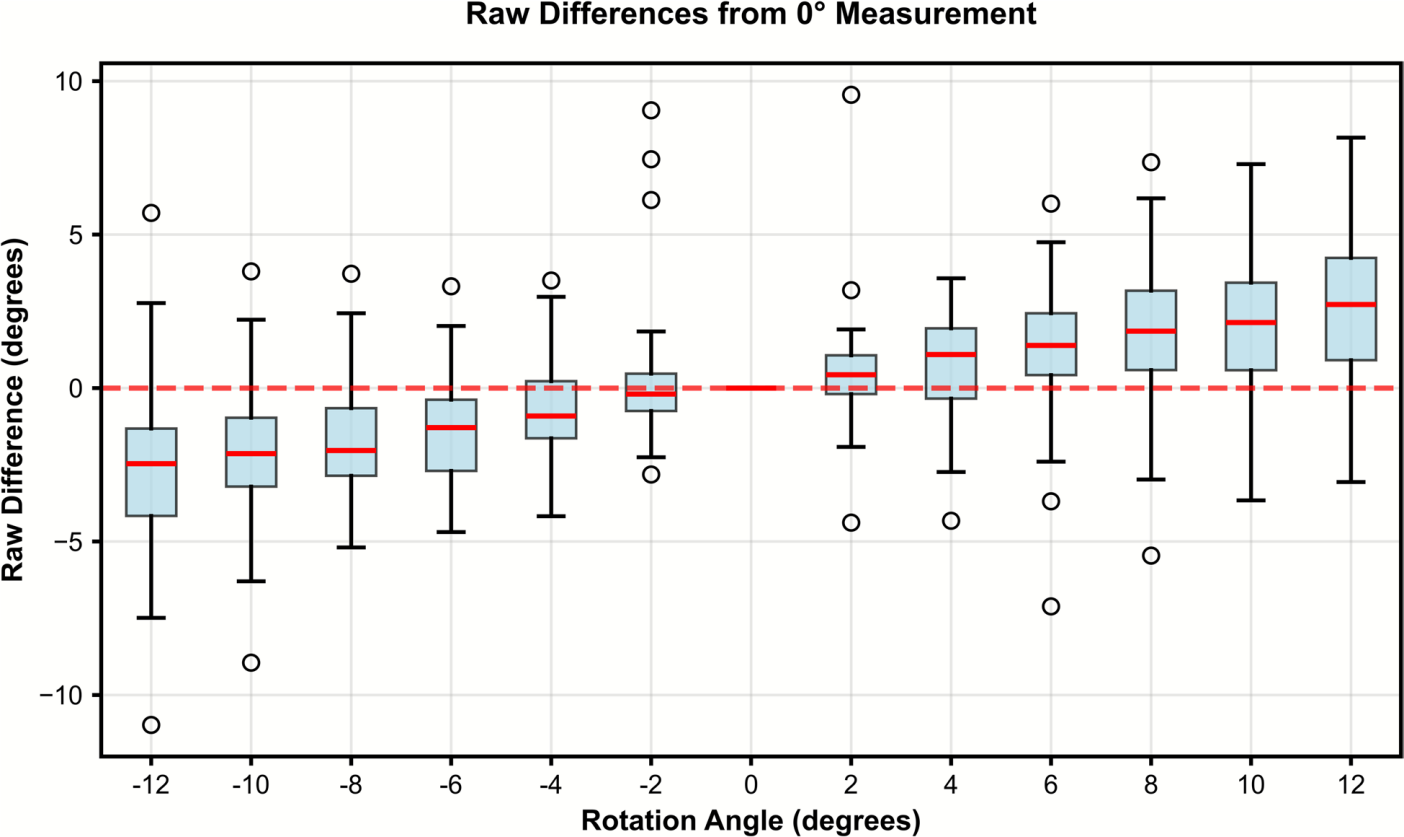
Effect of axial rotation on posterior tibial slope measurements

**Fig. 7:**
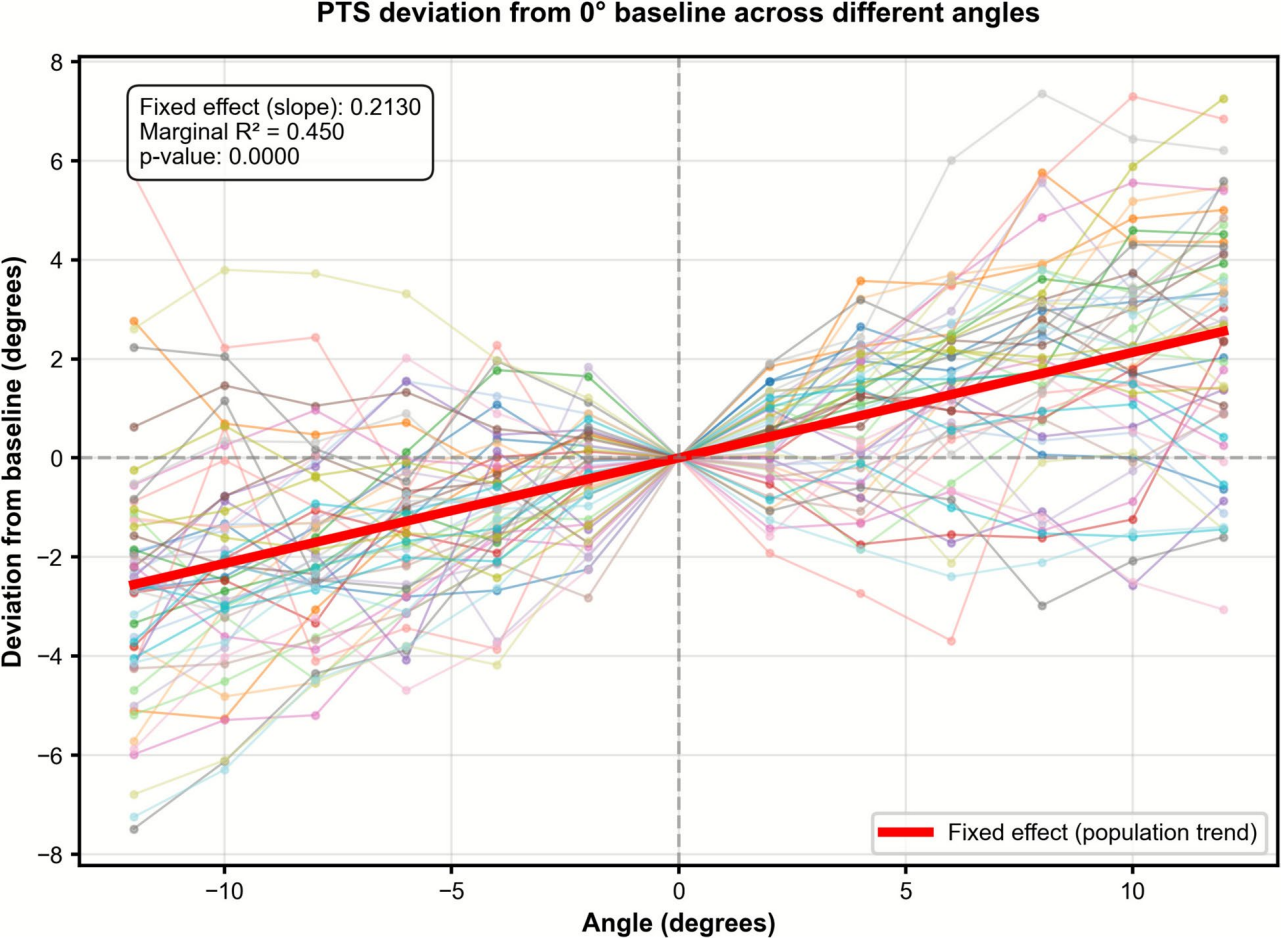
PTS measurement differences from baseline plotted against malrotation angle

PTS measurements increased significantly with greater internal axial malrotation. Measurements across all angles and patients (*n* = 55) are visualized and summarized in Figs. 6, 7. While the overall trend showed decreasing PTS with internal rotation and increasing PTS with external rotation, several knees exhibited small sign reversals around the 0° line (Fig. 6), likely due to subtle variability of automated joint line detection on rotated DRRs. As shown in Fig. 7, the linear mixed-effects model revealed a significant linear relationship between PTS measurement deviation and rotation angle, with a slope of 0.2° per 1° of rotation and a marginal *R*^2^ of 0.45 (*p* < 0.01). This indicates a predictable increase in measurement error with increasing malrotation, such that a 5° deviation results in approximately 1° of PTS error on average.

To complement this trend-based model, we performed a discrete-angle analysis to identify a practical threshold for clinically meaningful error. As shown in Table 2, malrotation of ≥ 6°—in either direction—resulted in statistically significant differences from baseline (*p* < 0.01) and exceeded a 1° mean absolute error.

**Table 2:** Average values and *t*-test results of PTS measurement deviations compared with true lateral

Rotated angle	Mean diff. (SD)	*p*-Value
−12°	−2.69 (2.7)	< 0.001
−10°	−2.14 (2.3)	< 0.001
−8°	−1.83 (1.9)	< 0.001
−6°	−1.38 (1.7)	< 0.001
−4°	−0.69 (1.7)	0.003
−2°	−0.09 (1.0)	0.705
2°	0.47 (1.7)	0.048
4°	0.68 (1.6)	0.002
6°	1.19 (2.1)	< 0.001
8°	1.79 (2.4)	< 0.001
10°	2.03 (2.4)	< 0.001
12°	2.55 (2.5)	< 0.001

**Fig. 8:**
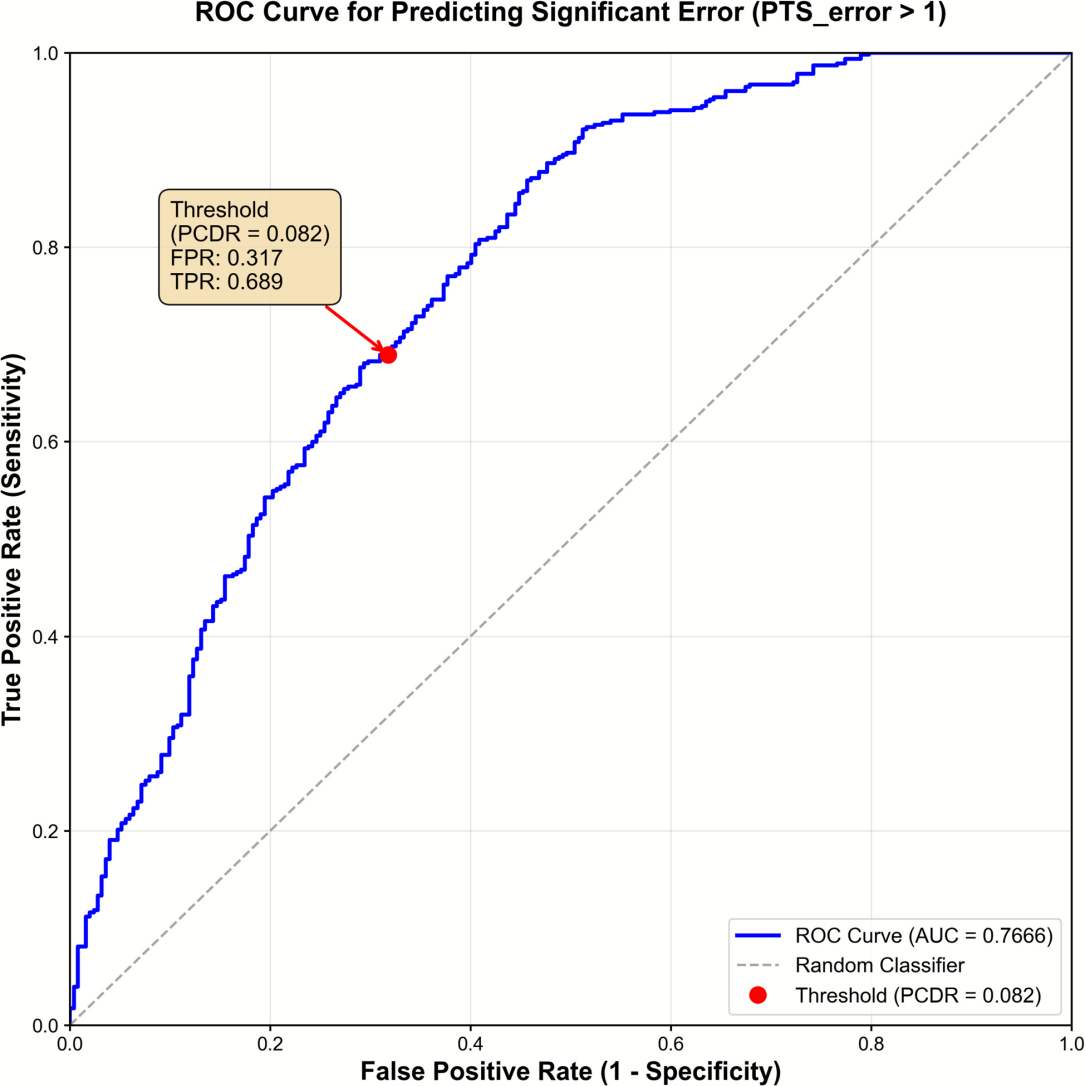
Receiver operating characteristic (ROC) curve of PCDR for detecting radiographs with > 1° PTS error

As expected from trigonometric projection, PCDR exhibited a near-linear relationship with applied malrotation angle (*R*^2^ > 0.98 for both internal and external rotation; Supplementary Fig. S4), confirming it as a quantitative marker of axial malrotation. On the basis of this relationship, a malrotation of ±6° mentioned above to produce > 1° PTS error corresponded to a PCDR of approximately 0.082. ROC analysis (Fig. 8) showed that PCDR achieved fair discriminative performance for identifying radiographs with > 1° PTS error (AUROC = 0.77).

The plot shows the difference between AI and human measurements against their mean. The dashed gray line indicates the mean difference (bias), and the red dashed lines represent the limits of agreement (± 1.96 standard deviations). The narrow bias and symmetric spread suggest good agreement between the AI model and expert measurements across the range of values.

The box plot compares posterior tibial slope (PTS) measurements at different femoral rotation angles relative to the neutral (0°) position. Raw differences show systematic directional bias: external rotation (negative angles) decreases measured PTS, while internal rotation (positive angles) increases it. The red dashed line indicates zero difference.

Deviation from baseline is plotted, with the linear mixed effects model visualized as thick red line. The linear effects model (red line) shows a linear relationship between PTS measurement deviation and rotation angle, with a slope of 0.2° per 1° of rotation and a marginal *R*^2^ of 0.45 (*p* < 0.01).

The red marker denotes the physiologically derived threshold (PCDR of 0.082), which corresponds to ±6° malrotation. At this threshold, the true positive rate was 0.689 and the false positive rate was 0.317.

## Discussion

We analyzed the effect of malrotation on PTS using real patient data to identify a consistent, predictable relationship. By combining DRR simulation and AI-based measurement, PTS measurements were obtained under controlled conditions in a larger and more anatomically diverse cohort than in previous studies. [12–14]. This study (1) has shown a consistent linear relationship between malrotation and PTS measurement error, and (2) suggests that malrotation of 6° or more—corresponding to a PCDR of approximately 0.082—results in PTS measurement errors exceeding 1°. The value of PCDR as a quality marker was supported by ROC analysis, where PCDR achieved an AUROC of 0.77 for identifying radiographs with > 1° PTS error, indicating fair discriminative performance.

### Systematic analysis

Our findings align with previous studies showing that posterior condylar distance increases with axial rotation and correlates with PTS error [9, 10]. While differences in measurement methods and patient anatomy limit direct comparison, our DRR-based estimates of malrotation and error closely parallel observed values from clinical radiographs.

Notably, our results were consistent with those of Mayer et al., who also reported an approximate 0.2° change in PTS per 1° of axial malrotation using a tibial saw bone model and posterior tibial condyle overlap as the reference for alignment [14]. While femoral condyle overlap is more commonly used for acquiring lateral knee radiographs, tibial-based alignment may offer anatomical advantages. However, our study aimed not to define an absolute “true” PTS, but to quantify how PTS measurements change relative to a fixed radiographic reference. Whether femoral or tibial condyle alignment is used simply shifts the baseline; the linear relationship between rotation and measurement error remains consistent.

Our study builds upon previous work by combining their strengths with novel methodology by analyzing a relatively large and anatomically diverse sample while precisely controlling the degree of malrotation through the use of DRRs from CT images. Interestingly, although the AI model used for PTS measurement was trained exclusively on real-world radiographs, it demonstrated good agreement when applied to DRRs (ICC = 0.73–0.82), comparable to its external validation performance on lateral radiographs from a separate institution (ICC = 0.73–0.80) [18]. This suggests that (1) cone-beam generated DRRs sufficiently replicate key radiographic features necessary for reliable anatomical analysis, and (2) AI models trained on large datasets of clinical radiographs may generalize effectively to simulated inputs.

### Clinical implications

Our findings have significant clinical implications, particularly for improving the quality assurance of lateral knee radiographs and for guiding slope-based surgical decision-making. Even modest axial malrotation can meaningfully alter the measured posterior tibial slope, with a 6° rotation producing an average 1° difference in apparent PTS. This magnitude of error is sufficient to influence procedures in which slope accuracy is critical—such as anterior cruciate ligament (ACL) reconstruction, slope-decreasing high tibial osteotomy, and total knee arthroplasty alignment planning. Moreover, while our simulations fixed varus/valgus and flexion/extension malalignment at zero, such positioning errors and deformities are common in daily practice and may further exacerbate the inaccuracy of radiographic PTS. This reinforces that, under nonideal conditions, PTS values from simple radiographs should be interpreted with caution.

Therefore, radiographs exceeding a malrotation threshold—such as the 6° cutoff identified in this study—should be carefully reviewed or reacquired to ensure reliable measurements. A simple PCDR calculation can serve as an immediate quantitative indicator of lateral image quality. In clinical workflows, this enables radiology teams to identify suboptimal radiographs before they are used for preoperative assessment, thereby preventing slope misinterpretation. While PCDR measurement on clinical radiographs now require a single manual step as shown in Supplementary Fig. S5, the methodology is feasible for fully automated implementation in picture archiving systems. By allowing objective real-time radiographic quality assessment [24, 25], this approach supports the integration of standardized imaging control into clinical workflows—a critical step toward improving musculoskeletal surgical planning and enhancing the robustness of AI-based tools [26–28].

### Broader methodological applications

The DRR–AI framework demonstrated strong technical validation with AI-based measurements on DRRs showing good agreement with expert annotations (ICC = 0.78), comparable to the model’s performance on real radiographs from independent institutions (ICC = 0.73–0.80) [18]. Therefore, our pipeline may also serve as a powerful platform for enhancing the development and reliability of AI-based radiographic tools. While the use of AI in musculoskeletal imaging has increased greatly [26, 28–30], AI measurements can be very prone to error caused by image quality variation. Our results demonstrate not only the ability to filter out low-quality images but the ability to generate synthetic “difficult” images with controlled malrotation or other acquisition errors—which can be used to train and validate AI models on underrepresented cases in clinical datasets [31, 32]. The ability of an AI model trained solely on clinical radiographs to perform on DRRs with similar reliability to clinical external validation further supports the feasibility of using simulated radiographs to stress-test and enhance AI robustness. This dual function of the DRR–AI pipeline, as both a gatekeeper and a data generator, offers a scalable approach for improving model robustness and generalizability in musculoskeletal imaging AI.

### Study limitations

Several limitations must be acknowledged in this proof-of-concept study. First, validation used DRRs rather than repeated clinical radiographs under varying malrotation. While direct clinical validation would be ideal, it is ethically and logistically infeasible to obtain systematic rotational variations from patients. The strong correlation between our simulation results and clinical observations supports this approach’s validity [14].

Also, while an overall trend of increasing PTS with internal rotation was observed, several knees showed crossing of the 0° reference line in Fig. 6, suggesting inconsistency with our hypothesis. This can be explained by Fig. 5, where PTS measurements by human and model mostly agree with an ICC of 0.78, and some knees show differences > 5°. This inaccuracy can be explained by the morphological changes near joint line with larger amounts of malrotation and lower resolution of DRRs making automatic PTS measurements difficult in some cases. Nevertheless, the overall linear relation between applied rotation and measured PTS remained consistent, confirming that these fluctuations represent local variability rather than systematic bias.

Third, we examined only axial malrotation, while coronal tilt of the tibial plateau—which can arise from varus/valgus deformity or abduction/adduction positioning errors—was fixed to zero in our aligned volume. We excluded this factor because even minor tibial tilt would cause both medial and lateral plateaus to appear in the sagittal view, thereby reducing the precision of automatic slope detection by the AI model. Although this alignment eliminated confounding from coronal orientation, such tilt may contribute to PTS measurement error, as shown by Bixby et al. [12].

Lastly, it should be noted that our cohort consisted of preoperative patients scheduled for total knee arthroplasty, selected on the basis of the availability of high-resolution CT scans. Although knees with severe deformities were excluded, this elderly population (mean age of 72 years) often exhibits degenerative changes, minor deformity, or osteoporotic bone quality. Therefore, the generalizability of our findings to younger patients with normal anatomy may be limited, and further validation in a larger, more diverse cohort with high-resolution imaging is warranted.

## Conclusions

Using simulated radiographs generated from CT scans, we found that PTS measurements become unreliable when knee radiographs are rotated more than 6°, with errors exceeding 1°. These findings provide a practical framework for interpreting radiographic PTS in daily clinical practice—highlighting that PTS values should always be evaluated in the context of image rotation and quality. By implementing straightforward quality control based on PCDR, clinicians can minimize rotation-induced measurement errors and make more reliable, anatomy-based surgical decisions.

## Supplementary Information

Supplementary Material 1.

## Data Availability

The datasets used and/or analyzed during the current study are available from the corresponding author on reasonable request.

## References

[1] Shelburne KB, Kim H-J, Sterett WI et al (2011) Effect of posterior tibial slope on knee biomechanics during functional activity. J Orthop Res 29:223–231 20857489 10.1002/jor.21242

[2] Ersin M, Demirel M, Civan M et al (2023) The effect of posterior tibial slope on anteroposterior stability in posterior cruciate retaining total knee arthroplasty. BMC Musculoskelet Disord 24:390 37194040 10.1186/s12891-023-06507-6 PMC10186777

[3] Liu Z, Jiang J, Yi Q et al (2022) An increased posterior tibial slope is associated with a higher risk of graft failure following ACL reconstruction: a systematic review. Knee Surg Sports Traumatol Arthrosc 30:2377–2387 35124715 10.1007/s00167-022-06888-6

[4] Fares A, Horteur C, Abou Al Ezz M et al (2023) Posterior tibial slope (PTS) ≥ 10 degrees is a risk factor for further anterior cruciate ligament (ACL) injury; BMI is not. Eur J Orthop Surg Traumatol 33:2091–2099 36201030 10.1007/s00590-022-03406-9 PMC10275806

[5] Okazaki Y, Furumatsu T, Hiranaka T et al (2021) Steep posterior slope of the medial tibial plateau is associated with ramp lesions of the medial meniscus and a concomitant anterior cruciate ligament injury. Asia Pac J Sports Med Arthrosc Rehabil Technol 24:23–28 33680859 10.1016/j.asmart.2021.01.005 PMC7896126

[6] Conyer RT, Allen TG, Reinholz AK et al (2024) Effect of posterior tibial slope on outcomes after posterior cruciate ligament reconstruction. Orthop J Sports Med 12:23259671241236804 38544875 10.1177/23259671241236804 PMC10966978

[7] Zhang Z-y, Hong L-j, Bai W-b et al (2024) Increased global posterior tibial slope is significantly associated with higher ACL graft signal intensity on 2-year postoperative MRI after primary ACL reconstruction using hamstring tendon autografts. BMC Musculoskelet Disord 25:905 39538228 10.1186/s12891-024-08032-6 PMC11559053

[8] Yazdi HR, Torkaman A, Ebrahimzadeh Babaki A et al (2023) Fixation method can affect posterior tibial slope in opening-wedge high tibial osteotomy: a retrospective study. J Orthop Surg Res 18:780 37848897 10.1186/s13018-023-04281-8 PMC10583342

[9] Guo N, Smith CR, Schütz P et al (2024) Posterior tibial slope influences joint mechanics and soft tissue loading after total knee arthroplasty. Front Bioeng Biotechnol 12:1352794 38686117 10.3389/fbioe.2024.1352794 PMC11056792

[10] Dejour H, Bonnin M (1994) Tibial translation after anterior cruciate ligament rupture. Two radiological tests compared. J Bone Joint Surg Br 76:745–749 8083263

[11] Gwinner C, Fuchs M, Sentuerk U et al (2019) Assessment of the tibial slope is highly dependent on the type and accuracy of the preceding acquisition. Arch Orthop Trauma Surg 139:1691–1697 31104087 10.1007/s00402-019-03201-y

[12] Bixby EC, Tedesco LJ, Confino JE et al (2023) Effects of malpositioning of the knee on radiographic measurements: the influence of adduction, abduction, and malrotation on measured tibial slope. Orthop J Sports Med 11:23259671231164670 37347024 10.1177/23259671231164670 PMC10280522

[13] Vieider RP, Mehl J, Rab P et al (2024) Malrotated lateral knee radiographs do not allow for a proper assessment of medial or lateral posterior tibial slope. Knee Surg Sports Traumatol Arthrosc 32:1462–1469 38629758 10.1002/ksa.12170

[14] Mayer P, Leiprecht J, Schlumberger M et al (2025) Malrotation strongly influences posterior tibial slope measurement on lateral radiographs of the knee. Orthop J Sports Med 13:23259671251330309 40376391 10.1177/23259671251330309 PMC12078948

[15] Wangler S, Hofmann J, Moser HL et al (2024) Image correlation between digitally reconstructed radiographs, C-arm fluoroscopic radiographs, and x-ray: a phantom study. Cureus 16:e51868 38327943 10.7759/cureus.51868 PMC10849007

[16] Fuller RM, Kim J, An TW et al (2022) Assessment of flatfoot deformity using digitally reconstructed radiographs: reliability and comparison to conventional radiographs. Foot Ankle Int 43:983–993 35590471 10.1177/10711007221089260

[17] Ryu B, Nam JW, Ro DH et al (2025) Automated posterior tibial slope measurement using lateral knee radiographs: a novel landmark-based approach using deep learning. Orthop J Sports Med 13:23259671251331067 40297052 10.1177/23259671251331067 PMC12034990

[18] Martin KR, Haaland S, Persson A et al (2025) External validation of a novel landmark-based deep learning automated tibial slope measurement algorithm applied on short radiographs obtained in patients with ACL injuries. Orthop J Sports Med 13:23259671251333607 40342354 10.1177/23259671251333607 PMC12056323

[19] Bellon MR, Siddiqui MS, Ryu S et al (2014) The effect of longitudinal CT resolution and pixel size (FOV) on target delineation and treatment planning in stereotactic radiosurgery. J Radiosurg SBRT 3:149–163 29296396 PMC5675487

[20] Wang S, Xiao Z, Lu Y et al (2021) Radiographic optimization of the lateral position of the knee joint aided by CT images and the maximum intensity projection technique. J Orthop Surg Res 16:581 34627301 10.1186/s13018-021-02740-8 PMC8501547

[21] van Aarle W, Palenstijn WJ, De Beenhouwer J et al (2015) The ASTRA toolbox: a platform for advanced algorithm development in electron tomography. Ultramicroscopy 157:35–47 26057688 10.1016/j.ultramic.2015.05.002

[22] Koo TK, Li MY (2016) A guideline of selecting and reporting intraclass correlation coefficients for reliability research. J Chiropr Med 15:155–163 27330520 10.1016/j.jcm.2016.02.012 PMC4913118

[23] Nakagawa S, Schielzeth H (2013) A general and simple method for obtaining R2 from generalized linear mixed-effects models. Methods Ecol Evol 4:133–142

[24] Sun H, Wang W, He F et al (2023) An AI-based image quality control framework for knee radiographs. J Digit Imaging 36:2278–2289 37268840 10.1007/s10278-023-00853-6 PMC10501977

[25] Wang Q, Han X, Song L et al (2024) Automatic quality assessment of knee radiographs using knowledge graphs and convolutional neural networks. Med Phys 51:7464–7478 39016559 10.1002/mp.17316

[26] Kijowski R, Liu F, Caliva F et al (2020) Deep learning for lesion detection, progression, and prediction of musculoskeletal disease. J Magn Reson Imaging 52:1607–1619 31763739 10.1002/jmri.27001 PMC7251925

[27] Ruitenbeek HC, Oei EHG, Visser JJ et al (2024) Artificial intelligence in musculoskeletal imaging: realistic clinical applications in the next decade. Skelet Radiol 53:1849–1868 10.1007/s00256-024-04684-6 38902420

[28] Guermazi A, Omoumi P, Tordjman M et al (2024) How AI may transform musculoskeletal imaging. Radiology 310:e230764 38165245 10.1148/radiol.230764 PMC10831478

[29] Fritz B, Fritz J (2022) Artificial intelligence for MRI diagnosis of joints: a scoping review of the current state-of-the-art of deep learning-based approaches. Skelet Radiol 51:315–329 10.1007/s00256-021-03830-8 PMC8692303 34467424

[30] Litjens G, Kooi T, Bejnordi BE et al (2017) A survey on deep learning in medical image analysis. Med Image Anal 42:60–88 28778026 10.1016/j.media.2017.07.005

[31] Gao C, Killeen BD, Hu Y et al (2023) Synthetic data accelerates the development of generalizable learning-based algorithms for X-ray image analysis. Nat Mach Intell 5:294–308 38523605 10.1038/s42256-023-00629-1 PMC10959504

[32] Sizikova E, Badal A, Delfino JG et al (2024) Synthetic data in radiological imaging: current state and future outlook. BJR|Artificial Intell. 10.1093/bjrai/ubae007 10.1093/bjrai/ubae007 PMC13045695 42064396

