# Supplementary material for: Effects of axial malrotation on posterior tibial slope measurement: a digitally reconstructed radiograph study enabling automated quality assessment
Source: Knee Surg Relat Res. 2026 Jan 27;38:3. doi: 10.1186/s43019-026-00303-x (PMC12849264; doi:10.1186/s43019-026-00303-x)
Supplement: Supplementary file 1 — Supplementary Material 1. [file 43019_2026_303_MOESM1_ESM.docx]

**1. CT Volume Alignment for True Lateral DRR**

First, anterior–posterior (AP) projections of the CT volume were generated to identify the medial and lateral tibial edges. The volume was then rotated to eliminate mediolateral tilt of the tibial plateau in the coronal plane (Figure S1).

Next, the volume was rotated on the axial plane to achieve overlap of the medial and lateral femoral condyles in lateral projection, following the steps below:

1) The 3D segmentation mask of the femur was split into medial and lateral parts, then projected to the sagittal plane to create 2D masks for each condyle.

2) The anterior cortical line was defined as the extension of a segment connecting the femoral notch and a point 5 cm proximal along the anterior femoral shaft. The farthest point from this line on each condyle was defined as the posterior point (Figure S2a).

3) 3D coordinates of medial and lateral posterior points of the medial and lateral condyles were found, and the volume was rotated so that when visualized on the axial plane, the line connecting medial and lateral posterior points would be parallel to the mediolateral axis. (Figure 2b-c)

**Figure S1. Rotation in coronal plane to align medial and lateral tibial plateaus**

**a)** Anterior-posterior projected DRR of original CT image, **b)** Anterior-posterior projected DRR of aligned CT image. Red dots are medial and lateral tibial edges, and green line connecting red dots visualize mediolateral tilt of tibial plateau. In b), the tibial plateau is perpendicular to the craniocaudal axis of CT image.

**Figure S2. Rotation in axial plane to align medial and lateral femoral condyles**

**a)** Calculation of posterior point on 2D segmentation mask. The anterior cortical line (red) and posterior point (bright green), and femoral notch and anterior shaft point (blue) are visualized. **b)** Axial slice of femur bone mask at femoral condyle of original CT image, **c)** Axial slice of femur bone mask at femoral condyle of aligned CT image. Red dots are most distal point of medial and lateral posterior condyles, and the green line connecting red dots represent tilt of femoral condyles. In b), line connecting femoral condyles are parallel to mediolateral axis of CT image.

**2. Automatic calculation of PCDR**

Posterior points of the medial and lateral components of the femur were calculated from 2D lateral segmentation masks using the method described in Supplementary Figure 2, using a common anterior cortical line derived from the full femur 2D mask but separate contours from each medial and lateral mask. The absolute distance between medial and lateral posterior points in respect to the common anterior cortical line of the full femur 2D mask was defined as posterior condyle distance. To account for variation in femur size and image spacing, the value was normalized by dividing it by the distance between anterior cortical line and posterior point from the full femur 2D mask, yielding posterior condyle distance ratio (PCDR).

**Figure S3. Automatic calculation of posterior femoral condyle distance from segmentation masks**

a, b): 2D medial and lateral femur masks and anterior cortical line (red) from full mask and posterior point (green) obtained from each mask. c) Contours of medial (red) and lateral (green) femur masks visualized on lateral DRR. The posterior femoral condyle distance (blue line) was normalized by dividing it by the total anterior-posterior width of femoral joint (sum of white and blue segments). In this image, posterior femoral condyle distance ratio was calculated as 0.17.

**Figure S4. Posterior condyle distance ratio plotted against malrotation angle.**

Each patient is plotted as a gray connected line. Blue and green dashed lines represent results from linear regression for external and internal malrotation, respectively.

**Figure S5. Demonstration of semi-automated tool for estimating axial malrotation in lateral knee radiographs**

Anterior cortical line of femur (blue line) and posterior condyle point (orange point) of the larger condyle is automatically calculated for a given input radiograph. When the user selects the posterior point of smaller condyle (bright green point), posterior condyle distance ratio is automatically measured with estimated malrotation angles, enabling rapid assessment.
